# Supplementary material for: Development of a computer-aided design software for the quantitative evaluation of aesthetic damage
Source: PLoS One. 2019 Dec 18;14(12):e0226322. doi: 10.1371/journal.pone.0226322 (PMC6919621; doi:10.1371/journal.pone.0226322)
Supplement: S1 Code site — 1. (ZIP) [file pone.0226322.s001.zip › [EESC_jr][Projeto]Relatorio_07_21/reports/templates_zip/gentelella-master/production/general_elements.html]

Gentellela Alela! | 


Gentellela Alela!

Welcome,

## John Doe

  


### General

- Home 
  - Dashboard
  - Dashboard2
  - Dashboard3
- Forms 
  - General Form
  - Advanced Components
  - Form Validation
  - Form Wizard
  - Form Upload
  - Form Buttons
- UI Elements 
  - General Elements
  - Media Gallery
  - Typography
  - Icons
  - Glyphicons
  - Widgets
  - Invoice
  - Inbox
  - Calendar
- Tables 
  - Tables
  - Table Dynamic
- Data Presentation 
  - Chart JS
  - Chart JS2
  - Moris JS
  - ECharts
  - Other Charts
- Layouts 
  - Fixed Sidebar
  - Fixed Footer

### Live On

- Additional Pages 
  - E-commerce
  - Projects
  - Project Detail
  - Contacts
  - Profile
- Extras 
  - 403 Error
  - 404 Error
  - 500 Error
  - Plain Page
  - Login Page
  - Pricing Tables
- Multilevel Menu 
  - Level One- Level One
      - Level Two
      - Level Two
      - Level Two
    - Level One
- Landing Page Coming Soon

- John Doe
  - Profile
  - 50%
    Settings
  - Help
  - Log Out
- 6
  - John Smith
    3 mins ago

    Film festivals used to be do-or-die moments for movie makers. They were where...
  - John Smith
    3 mins ago

    Film festivals used to be do-or-die moments for movie makers. They were where...
  - John Smith
    3 mins ago

    Film festivals used to be do-or-die moments for movie makers. They were where...
  - John Smith
    3 mins ago

    Film festivals used to be do-or-die moments for movie makers. They were where...
  - **See All Alerts**


### General Elements

Go!

## Tabs Float left

- - Settings 1
  - Settings 2

- Home
- Profile
- Profile

Raw denim you probably haven't heard of them jean shorts Austin. Nesciunt tofu stumptown aliqua, retro synth master cleanse. Mustache cliche tempor, williamsburg carles vegan helvetica. Reprehenderit butcher retro keffiyeh dreamcatcher
synth. Cosby sweater eu banh mi, qui irure terr.

Food truck fixie locavore, accusamus mcsweeney's marfa nulla single-origin coffee squid. Exercitation +1 labore velit, blog sartorial PBR leggings next level wes anderson artisan four loko farm-to-table craft beer twee. Qui photo
booth letterpress, commodo enim craft beer mlkshk aliquip

xxFood truck fixie locavore, accusamus mcsweeney's marfa nulla single-origin coffee squid. Exercitation +1 labore velit, blog sartorial PBR leggings next level wes anderson artisan four loko farm-to-table craft beer twee. Qui photo
booth letterpress, commodo enim craft beer mlkshk

## Tabs Float right

- - Settings 1
  - Settings 2

- Home
- Profile
- Profile

Raw denim you probably haven't heard of them jean shorts Austin. Nesciunt tofu stumptown aliqua, retro synth master cleanse. Mustache cliche tempor, williamsburg carles vegan helvetica. Reprehenderit butcher retro keffiyeh dreamcatcher
synth. Cosby sweater eu banh mi, qui irure terr.

Food truck fixie locavore, accusamus mcsweeney's marfa nulla single-origin coffee squid. Exercitation +1 labore velit, blog sartorial PBR leggings next level wes anderson artisan four loko farm-to-table craft beer twee. Qui photo
booth letterpress, commodo enim craft beer mlkshk aliquip

xxFood truck fixie locavore, accusamus mcsweeney's marfa nulla single-origin coffee squid. Exercitation +1 labore velit, blog sartorial PBR leggings next level wes anderson artisan four loko farm-to-table craft beer twee. Qui photo
booth letterpress, commodo enim craft beer mlkshk

## Vertical Tabs Float left

- - Settings 1
  - Settings 2

- Home
- Profile
- Messages
- Settings

Home tab

Raw denim you probably haven't heard of them jean shorts Austin. Nesciunt tofu stumptown aliqua, retro synth master cleanse. Mustache cliche tempor, williamsburg carles vegan helvetica. Reprehenderit butcher retro keffiyeh dreamcatcher
synth. Cosby sweater eu banh mi, qui irure terr.

Profile Tab.

Messages Tab.

Settings Tab.

## Vertical Tabs Float right

- - Settings 1
  - Settings 2

Home tab

Raw denim you probably haven't heard of them jean shorts Austin. Nesciunt tofu stumptown aliqua, retro synth master cleanse. Mustache cliche tempor, williamsburg carles vegan helvetica. Reprehenderit butcher retro keffiyeh dreamcatcher
synth. Cosby sweater eu banh mi, qui irure terr.

Profile Tab.

Messages Tab.

Settings Tab.

- Home
- Profile
- Messages
- Settings

## Collapsible / Accordion Sessions

- - Settings 1
  - Settings 2

#### Collapsible Group Items #1

| # | First Name | Last Name | Username |
| --- | --- | --- | --- |
| 1 | Mark | Otto | @mdo |
| 2 | Jacob | Thornton | @fat |
| 3 | Larry | the Bird | @twitter |

#### Collapsible Group Items #2

**Collapsible Item 2 data**

Anim pariatur cliche reprehenderit, enim eiusmod high life accusamus terry richardson ad squid. 3 wolf moon officia aute, non cupidatat skateboard dolor brunch. Food truck quinoa nesciunt laborum eiusmod. Brunch 3 wolf moon tempor,

#### Collapsible Group Items #3

**Collapsible Item 3 data**

Anim pariatur cliche reprehenderit, enim eiusmod high life accusamus terry richardson ad squid. 3 wolf moon officia aute, non cupidatat skateboard dolor brunch. Food truck quinoa nesciunt laborum eiusmod. Brunch 3 wolf moon tempor

## Collapsible / Accordion Sessions

- - Settings 1
  - Settings 2

#### Collapsible Group Item #1

| # | First Name | Last Name | Username |
| --- | --- | --- | --- |
| 1 | Mark | Otto | @mdo |
| 2 | Jacob | Thornton | @fat |
| 3 | Larry | the Bird | @twitter |

#### Collapsible Group Item #2

**Collapsible Item 2 data**

Anim pariatur cliche reprehenderit, enim eiusmod high life accusamus terry richardson ad squid. 3 wolf moon officia aute, non cupidatat skateboard dolor brunch. Food truck quinoa nesciunt laborum eiusmod. Brunch 3 wolf moon tempor,

#### Collapsible Group Item #3

**Collapsible Item 3 data**

Anim pariatur cliche reprehenderit, enim eiusmod high life accusamus terry richardson ad squid. 3 wolf moon officia aute, non cupidatat skateboard dolor brunch. Food truck quinoa nesciunt laborum eiusmod. Brunch 3 wolf moon tempor

## Tooltips Hover to view

- - Settings 1
  - Settings 2

Add small overlays of content for housing secondary information.

Left

Top

Bottom

Right

## Tooltips Hover to view

- - Settings 1
  - Settings 2

Hover over the links below to see tooltips:

Tooltip left
Tooltip top
Tooltip bottom
Tooltip right

## Daily active users Sessions

- - Settings 1
  - Settings 2

- Entertainment

  ## Who Needs Sundance When You’ve Got Crowdfunding?

  13 hours ago by Jane Smith

  Film festivals used to be do-or-die moments for movie makers. They were where you met the producers that could fund your project, and if the buyers liked your flick, they’d pay to Fast-forward and… Read More
- Entertainment

  ## Who Needs Sundance When You’ve Got Crowdfunding?

  13 hours ago by Jane Smith

  Film festivals used to be do-or-die moments for movie makers. They were where you met the producers that could fund your project, and if the buyers liked your flick, they’d pay to Fast-forward and… Read More
- Entertainment

  ## Who Needs Sundance When You’ve Got Crowdfunding?

  13 hours ago by Jane Smith

  Film festivals used to be do-or-die moments for movie makers. They were where you met the producers that could fund your project, and if the buyers liked your flick, they’d pay to Fast-forward and… Read More

## Daily active users Sessions

- - Settings 1
  - Settings 2

# Hello, world!

This is a simple hero unit, a simple jumbotron-style component for calling extra attention to featured content or information.

## Dropdowns Multiple dropdown designs

- - Settings 1
  - Settings 2

Gentellela provides you with several dropdown designs for your choosing.

- Regular link
- Dropdown
  - Action
  - Another action
  - Something else here
  - Separated link
- Dropdown
  - Action
  - Another action
  - Something else here
  - Separated link
- Dropdown
  - Action
  - Another action
  - Something else here
  - Separated link

## Modals

- - Settings 1
  - Settings 2

Large modal

×

#### Modal title

#### Text in a modal

Praesent commodo cursus magna, vel scelerisque nisl consectetur et. Vivamus sagittis lacus vel augue laoreet rutrum faucibus dolor auctor.

Aenean lacinia bibendum nulla sed consectetur. Praesent commodo cursus magna, vel scelerisque nisl consectetur et. Donec sed odio dui. Donec ullamcorper nulla non metus auctor fringilla.

Close
Save changes

Small modal

×

#### Modal title

#### Text in a modal

Praesent commodo cursus magna, vel scelerisque nisl consectetur et. Vivamus sagittis lacus vel augue laoreet rutrum faucibus dolor auctor.

Aenean lacinia bibendum nulla sed consectetur. Praesent commodo cursus magna, vel scelerisque nisl consectetur et. Donec sed odio dui. Donec ullamcorper nulla non metus auctor fringilla.

Close
Save changes

## Notifications Styled & Custom notifications

- - Settings 1
  - Settings 2

Regular notifications

Success
Info
Notice
Error
Dark


---

Sticky notifications.. these wont close unless user closes them.

Success
Info
Warning
Error
Dark


---

Non-Blocking Notice


---

Success
Info
Warning
Error
Dark

## Pop Overs Sessions

- - Settings 1
  - Settings 2

×
**Holy guacamole!** Best check yo self, you're not looking too good.

×
**Holy guacamole!** Best check yo self, you're not looking too good.

×
**Holy guacamole!** Best check yo self, you're not looking too good.

×
**Holy guacamole!** Best check yo self, you're not looking too good.

## Progress barSessions

- - Settings 1
  - Settings 2

Bootstrap progress bar with animated loading bar, add right to .progress to align right

## To Do List Sample tasks

- - Settings 1
  - Settings 2

- Schedule meeting with new client
- Create email address for new intern
- Have IT fix the network printer
- Copy backups to offsite location
- Food truck fixie locavors mcsweeney
- Food truck fixie locavors mcsweeney

## Daily active users Sessions

- - Settings 1
  - Settings 2

- John Smith
  3 mins ago

  Film festivals used to be do-or-die moments for movie makers. They were where you met the producers that
- John Smith
  3 mins ago

  Film festivals used to be do-or-die moments for movie makers. They were where you met the producers that
- John Smith
  3 mins ago

  Film festivals used to be do-or-die moments for movie makers. They were where you met the producers that
- John Smith
  3 mins ago

  Film festivals used to be do-or-die moments for movie makers. They were where you met the producers that


Gentelella - Bootstrap Admin Template by Colorlib
